# C-Reactive Protein-Based Composite Indices for Predicting Tumor Overgrowth Restenosis After Partially Covered Duodenal Stenting in Gastric Cancer: A Cohort Study

**DOI:** 10.3390/cancers18172803

**Published:** 2026-08-28

**Authors:** Hyuk Lee, Young Eun Oh, Tae-Se Kim, Yang Won Min, Byung-Hoon Min, Jun Haeng Lee

**Affiliations:** 1 Department of Medicine, Samsung Medical Center, Sungkyunkwan University School of Medicine, 81 Irwon-ro, Gangnam-gu, Seoul 06351, Republic of Korea; 2 Samsung Precision Genome Medicine Institute, Samsung Medical Center, 81 Irwon-ro, Gangnam-gu, Seoul 06351, Republic of Korea

**Keywords:** C-reactive protein, gastric outlet obstruction, gastric cancer, stents, tumor overgrowth restenosis

## Abstract

Background/Objectives: Partially covered duodenal stents rapidly relieve malignant gastric outlet obstruction, but tumor overgrowth restenosis limits durability. We compared six preprocedural inflammatory, nutritional, and immune indices for predicting this outcome in patients with gastric cancer. Methods: We retrospectively analyzed 68 consecutive patients from a prospectively maintained cohort. The primary endpoint was endoscopically or radiologically confirmed tumor overgrowth restenosis. Discrimination was assessed using receiver operating characteristic curves (pairwise DeLong tests with Bonferroni correction) and time-dependent areas under the curve (AUCs) accounting for death as a competing risk. Multivariable cause-specific Cox models were restricted to preprocedural covariates; post-stenting chemotherapy or radiotherapy was examined in time-dependent sensitivity analyses. Results: Technical and clinical success rates were 100% and 94.1%. Seventeen patients (25.0%) developed restenosis at a median of 66 days. The C-reactive protein–albumin–lymphocyte (CALLY) index showed the highest AUC (0.859), followed by the C-reactive protein-to-albumin ratio (CAR; 0.822) and neutrophil-to-lymphocyte ratio (0.774); these three indices did not differ significantly. At an exploratory, internally derived cutoff of ≤0.110, CALLY had 76.5% sensitivity and 84.3% specificity and remained associated with restenosis after adjustment for stenosis site and stent length (adjusted hazard ratio, 12.30; 95% confidence interval, 3.89–38.83; C-index, 0.836), with consistent results in continuous, time-dependent, and tumor-covariate-adjusted analyses. The 180-day cumulative incidence was 52.4% with low CALLY versus 4.3% with high CALLY. Conclusions: C-reactive protein-based indices showed the highest numerical discrimination, although pairwise differences among CALLY, CAR, and NLR were not statistically significant. CALLY is a promising exploratory biomarker for restenosis risk stratification, but its cutoff should not guide clinical decisions until externally validated.

## Simple Summary

Malignant gastric outlet obstruction is a common and debilitating complication of advanced gastric cancer that prevents adequate oral intake and substantially impairs quality of life. Endoscopic placement of a partially covered self-expandable metal stent restores luminal patency, but tumor overgrowth at the uncovered stent ends can cause recurrent obstruction. In 68 consecutive patients treated with a partially covered duodenal stent, we assessed whether six inflammatory, nutritional, and immune indices derived from routine preprocedural blood tests could identify those at higher risk of this complication. Tumor overgrowth restenosis developed in 17 patients (25%) a median of 66 days after stenting. Indices incorporating C-reactive protein, particularly the C-reactive protein-albumin-lymphocyte index, showed the highest numerical discrimination between patients who did and did not develop restenosis, although differences among the top-performing indices were not statistically significant. Because these tests are inexpensive and already part of routine care, they could eventually help clinicians recognize patients at higher risk of restenosis. Whether using such markers to guide surveillance or treatment actually improves outcomes has not been tested, however, and the proposed cutoff must be confirmed in larger, independent, multicenter studies before it is applied in practice.

## 1. Introduction

Malignant gastric outlet obstruction (GOO) is a debilitating complication of advanced gastric cancer [1]. In patients with unresectable disease, restoring luminal patency is essential for symptom relief, nutritional maintenance, and tolerance of systemic therapy. Endoscopic placement of a self-expandable metallic stent is widely used for palliation and, compared with surgical gastrojejunostomy, offers shorter procedure time, earlier resumption of oral intake, lower morbidity, and a shorter hospital stay [2,3,4]. Although endoscopic ultrasound (EUS)-guided gastroenterostomy is a highly effective alternative at specialized centers, enteral stenting remains widely applicable, particularly with limited life expectancy or anatomy unsuitable for this approach [5,6,7].

Enteral stenting is limited by two contrasting modes of failure: fully covered stents are prone to migration, whereas uncovered stents are susceptible to tumor ingrowth or overgrowth [8,9,10,11]. The partially covered design was developed to address both issues [12,13], but tumor overgrowth has emerged as the predominant pattern of late stent failure. Readily available predictors of stent patency could help clinicians identify high-risk patients and guide follow-up strategies.

Systemic inflammation and nutritional status are increasingly recognized as important determinants of outcomes in gastric cancer, and several indices derived from routine blood tests have been investigated as prognostic biomarkers, including the neutrophil-to-lymphocyte ratio (NLR), systemic immune-inflammation index (SII), prognostic nutritional index (PNI), modified Glasgow prognostic score (mGPS), and C-reactive protein (CRP)–albumin ratio (CAR) [14,15,16,17,18]. More recently, the CRP–albumin–lymphocyte index (CALLY) has been proposed as a composite biomarker integrating systemic inflammation, nutrition, and immune status [19]. Evidence for this index has been derived largely from patients undergoing curative resection or systemic chemotherapy [20,21]. In gastric cancer specifically, a low CALLY index has been linked to worse survival after gastrectomy and during palliative systemic therapy [22,23,24]. However, its prognostic value in palliative enteral stenting for malignant GOO remains insufficiently characterized.

Therefore, we evaluated the incidence and timing of tumor overgrowth restenosis after partially covered duodenal stent (PCDS) placement for malignant GOO and compared the prognostic performance of six preprocedural inflammatory, nutritional, and immunological indices—SII, NLR, CAR, PNI, mGPS, and CALLY—for predicting this outcome.

## 2. Materials and Methods

### 2.1. Patients and Study Design

We retrospectively analyzed a prospectively maintained cohort of consecutive patients who underwent PCDS placement for malignant GOO secondary to gastric adenocarcinoma at Samsung Medical Center, Seoul, between January 2021 and January 2024. Inclusion criteria were (1) clinical features of GOO with reduced oral intake; (2) obstruction caused by a primary malignant distal gastric tumor; (3) stenosis between the gastric corpus and duodenal bulb; and (4) unresectable disease or unsuitability for surgery. Exclusion criteria were mild obstruction controlled with a liquid diet, suspected perforation or peritonitis, multiple small-bowel obstructions due to peritoneal carcinomatosis, prior gastrojejunostomy or stenting, clinically significant active infection or sepsis at sampling, hematologic disorders, or recent granulocyte colony-stimulating factor or systemic corticosteroid use. Written informed consent was obtained from all patients. The study was approved by the Institutional Review Board of Samsung Medical Center (protocol SMC 2018-12-008-003) and conducted in accordance with the Declaration of Helsinki. This study is reported in accordance with the STROBE (Strengthening the Reporting of Observational Studies in Epidemiology) statement for cohort studies (Table S11).

### 2.2. Data Collection and Definitions

Baseline variables collected prospectively included age, sex, Eastern Cooperative Oncology Group (ECOG) performance status, stenosis location (antrum vs. gastric outlet/duodenal bulb), Gastric Outlet Obstruction Scoring System (GOOSS) scores at baseline and 72 h, and stent length. Tumor-related characteristics were also collected from the medical records: clinical M stage (the presence of distant metastasis) and sites of metastasis (liver, peritoneum, distant lymph nodes, lung); histologic differentiation (well, moderately, or poorly differentiated, or signet-ring cell carcinoma); Lauren classification; measured stricture length; concomitant biliary obstruction; and systemic chemotherapy given before stenting, including the number of prior treatment lines. Because no patient underwent resection, pathologic T and N stages could not be determined, and staging was therefore limited to the clinical M category. Chemotherapy or radiotherapy started after stenting was recorded as a postprocedural treatment variable, together with its start date. All stents had a uniform 20 mm body diameter and 40 mm proximal flare; therefore, stent diameter was not analyzed as a covariate.

Obstruction severity was assessed using the GOOSS (0, no oral intake; 1, liquids only; 2, soft solids; and 3, low-residue or normal diet). Technical success was defined as successful placement with complete expansion across the stenosis. Clinical success was defined as relief of obstructive symptoms and/or at least a one-grade improvement in GOOSS within 72 h [2].

The primary endpoint was tumor overgrowth restenosis, defined as endoscopically and/or radiologically confirmed luminal stenosis within or adjacent to the stent caused by tumor growth at an uncovered segment and accompanied by recurrent obstructive symptoms. Stent migration and all-cause mortality were recorded as separate events. Time to restenosis was calculated from stent placement. In cause-specific Cox analyses, patients without restenosis were censored at death or last follow-up; in cumulative incidence and Fine-Gray analyses, death without restenosis was treated as a competing event. Patients were followed at 1, 3, and 6 months and every 3 months thereafter, with additional visits when symptoms recurred. Cross-sectional imaging and/or endoscopy was performed when restenosis was suspected. Tumor overgrowth was distinguished from tumor ingrowth—defined as tumor extension through the stent mesh into the lumen—food impaction, stent migration, stent deformation, and extrinsic compression using endoscopic findings and fluoroscopic contrast studies, supplemented by contrast-enhanced cross-sectional imaging when needed. Overgrowth was diagnosed only when fluoroscopic contrast studies, performed in all suspected cases, showed contrast flowing freely through the stent lumen, without narrowing or delay within the stent body, and the luminal compromise was confined to the proximal and/or distal stent ends, where neoplastic tissue extending over an uncovered margin caused narrowing accompanied by recurrent obstructive symptoms. All restenosis events were adjudicated by two endoscopists experienced in enteral stenting for malignant strictures, and disagreements were resolved by consensus.

### 2.3. Stent and Procedure

**Figure 1:**
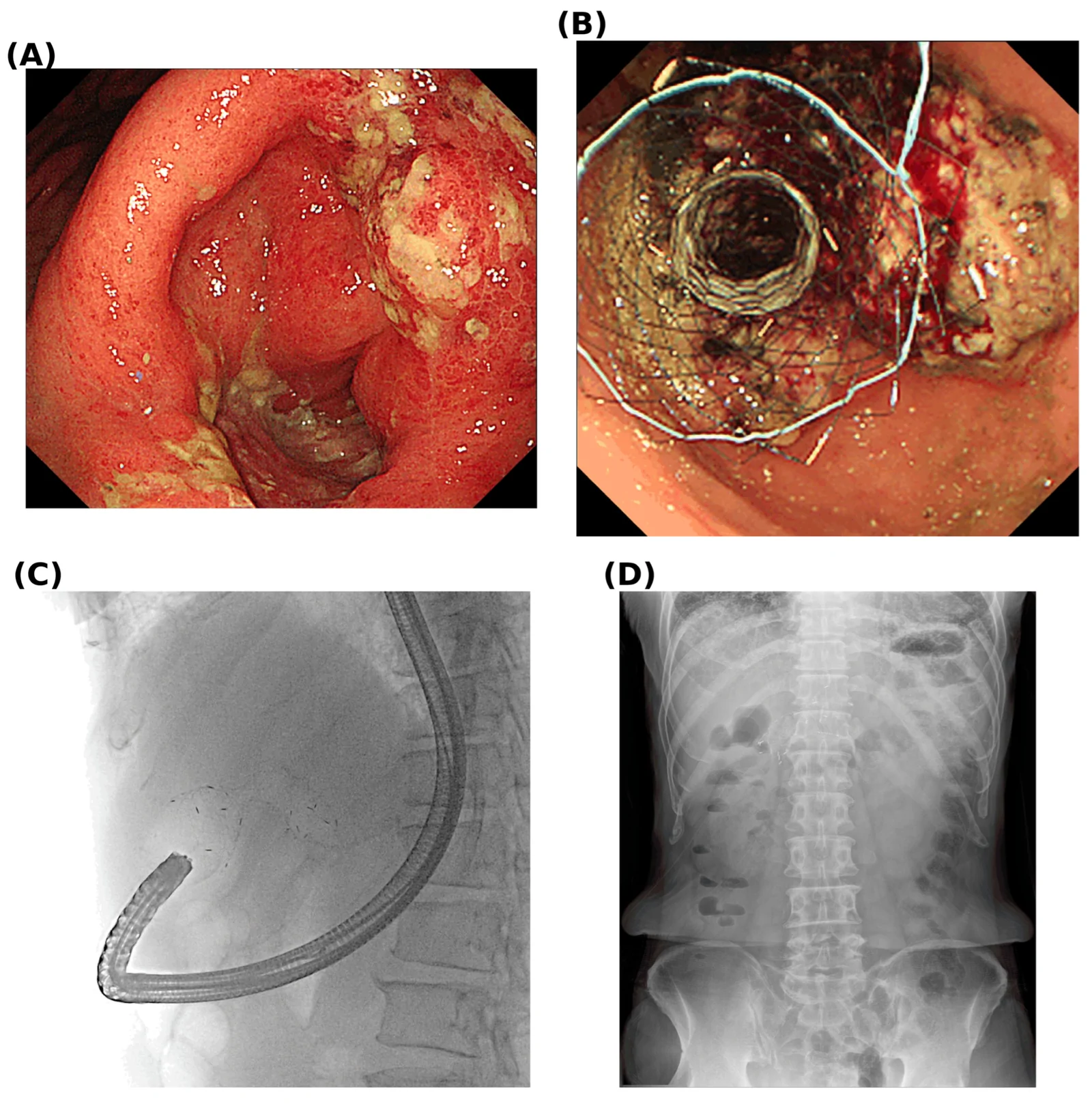
Endoscopic and fluoroscopic images of partially covered duodenal stent placement. Representative images of partially covered duodenal stent (HANAROSTENT Duodenum/Pylorus Kim’s Flare [NCN]; M.I.Tech) placement for malignant gastric outlet obstruction caused by gastric adenocarcinoma. (**A**) Severe luminal stenosis of the gastric outlet before stenting. (**B**) Proximal flare of the partially covered stent spanning the gastric outlet immediately after deployment. (**C**) Fluoroscopic image during deployment showing the radiopaque markers and stent outline across the malignant stenosis. (**D**) Abdominal radiograph obtained within 48 h after stenting, confirming stent expansion and restoration of luminal patency.

The PCDS (HANAROSTENT Duodenum/Pylorus Kim’s Flare [NCN]; M.I.Tech Co., Ltd., Pyeongtaek, Republic of Korea) is a twin-layer braided nitinol stent with a 20 mm body, a 40 mm proximal flare for the gastric outlet, and uncovered proximal and distal ends to enhance fixation. Usable lengths range from 60 to 150 mm. Procedures were performed under conscious sedation by an experienced endoscopist; patients with suspected biliary obstruction underwent biliary drainage beforehand. Stent length was selected to exceed the stenosis by at least 20 mm. After a guidewire crossed the obstruction, the stent was deployed under combined endoscopic and fluoroscopic guidance, with the proximal flare spanning the outlet (Figure 1). Immediate balloon dilation was performed if expansion was inadequate. Complete expansion was confirmed by abdominal radiography within 48 h, and oral intake was resumed gradually.

### 2.4. Laboratory Measurements and Indices

Peripheral blood samples were drawn before stenting (median, 2.5 days; range, 0–5 days) after at least 8 h of fasting; none were obtained after the procedure. Two patients were receiving antibiotics for mild bronchopneumonia without sepsis or hemodynamic compromise and were retained in the cohort; none had received systemic corticosteroids or granulocyte colony-stimulating factor. No patient received a large-volume intravenous fluid bolus immediately before blood sampling. From baseline complete blood count with differential, serum albumin, and CRP, the following six indices were calculated: (1) SII = neutrophil × platelet/lymphocyte; (2) NLR = neutrophil/lymphocyte; (3) CAR = CRP (mg/dL)/albumin (g/dL); (4) PNI = 10 × albumin + 0.005 × lymphocyte count; (5) mGPS, scored as 0 (CRP ≤ 1.0 mg/dL), 1 (CRP > 1.0 mg/dL and albumin ≥ 3.5 g/dL), or 2 (CRP > 1.0 mg/dL and albumin < 3.5 g/dL); and (6) CALLY = [albumin × lymphocyte]/[CRP × 10^4^] [19]. Lower CALLY values reflect greater systemic inflammation, malnutrition, and lymphocyte-mediated immune dysfunction. All indices were analyzed as baseline variables.

### 2.5. Statistical Analysis

Continuous variables were summarized as medians and interquartile ranges (IQRs). Because none followed a normal distribution according to the Shapiro–Wilk test (PROC UNIVARIATE), groups were compared using the Wilcoxon rank-sum test (PROC NPAR1WAY). Categorical variables were compared using Fisher’s exact test or the chi-square test (PROC FREQ), as appropriate. Wilson score 95% confidence intervals were calculated for sensitivity, specificity, and predictive values.

Discriminatory performance was assessed using receiver-operating characteristic (ROC) curves (PROC LOGISTIC), with area under the curve (AUC) and 95% confidence interval (CI) by the DeLong method. Exploratory cutoffs for continuous indices were derived from the Youden index; for mGPS, the clinical cutoff of 0 vs. ≥1 was used. Optimism in the apparent discriminatory performance of CALLY was assessed using 1000 bootstrap resamples with the 0.632+ method; this analysis quantified optimism in the AUC and did not constitute validation of the cutoff itself. Pairwise AUC comparisons used the DeLong method with Bonferroni correction across 15 comparisons. Time-dependent AUCs at 90 and 180 days were also estimated, using inverse-probability-of-censoring weighting with death treated as a competing risk and patients who died without restenosis retained as controls; 95% CIs were derived from 1000 bootstrap resamples.

Time to restenosis was analyzed within a competing-risk framework. Cumulative incidence functions (CIFs) of restenosis at 90 and 180 days were estimated using the Aalen–Johansen method (PROC LIFETEST with the EVENTCODE option), treating death without restenosis as a competing event; 95% CIs were obtained from 2000 bootstrap resamples, and equality of CIFs between strata was compared using Gray’s test. Cause-specific hazard ratios (HRs) were estimated using Cox models (PROC PHREG), with death without restenosis treated as a censoring event. Because the six indices were highly correlated (Spearman ρ = 0.30–0.93, PROC CORR), six separate multivariable models were fitted, each restricted to covariates available at the time of stenting so that immortal-time bias could not arise: one dichotomized index, stenosis location, and stent length. Stent length was retained because of its univariable association with restenosis and because it closely tracks stricture length and local tumor burden (Spearman ρ = 0.93 with measured stricture length). Post-stenting chemotherapy/radiotherapy was examined in two sensitivity analyses: as a time-fixed covariate replicating the originally prespecified models (Table S6), and as a time-dependent covariate updated at treatment initiation (Table S7). Further sensitivity analyses added tumor-related covariates and performance status (clinical M1 status, peritoneal metastasis, poorly differentiated or signet-ring cell histology, prior chemotherapy, and ECOG ≥ 3) to the CALLY-based model one at a time, and replaced stent length with measured stricture length (Table S8). To limit the information lost by dichotomization, each index was also analyzed as a continuous variable (log-transformed where right-skewed, per one standard deviation), with nonlinearity assessed using restricted cubic splines with four knots (Table S9; Figure S1). Model discrimination was assessed using Harrell’s C-index. For mGPS, complete separation prevented reliable HR estimation; only the Gray’s test *p*-value was reported. The proportional hazards assumption was verified by log-minus-log plots. As a sensitivity analysis, a univariable Fine–Gray subdistribution hazards model was fitted for CALLY. With only 17 events, the primary models were limited to three parameters (roughly 5.7 events per parameter), and all multivariable estimates should be regarded as exploratory. Primary analyses were performed with SAS version 9.4 (SAS Institute Inc., Cary, NC, USA); the time-dependent, spline, and bootstrap confidence-interval analyses used the lifelines library (version 0.30) in Python (version 3.10). Two-sided *p* < 0.05 was considered statistically significant.

## 3. Results

### 3.1. Patient Characteristics and Procedural Outcomes

Sixty-eight consecutive patients were enrolled (Table 1; Figure S2). The median age was 60 years, and 46 (67.6%) were male. The obstruction site was the antrum in 25 patients (36.8%) and the gastric outlet or duodenal bulb in 43 (63.2%); 32 patients (47.1%) received chemotherapy or radiotherapy after stenting. Distant metastasis (clinical M1) was present in 23 patients (33.8%): liver in 5, peritoneum in 10, distant lymph nodes in 7, and lung in 1. Thirty-nine patients (57.4%) had poorly differentiated or signet-ring cell histology, 24 (35.3%) had received systemic chemotherapy before stenting (1–8 prior treatment lines), and 3 (4.4%) had concomitant biliary obstruction. The median stricture length was 6.5 cm (interquartile range, 5.0–8.2).

Technical success was achieved in all 68 patients (100%), and clinical success at 72 h was achieved in 64 (94.1%). The remaining four patients tolerated a soft diet within 7 days. No procedure-related perforation, major bleeding, or in-hospital mortality occurred.

### 3.2. Follow-Up, Tumor Overgrowth Restenosis, and Survival

The median follow-up duration was 347 days (IQR, 146–566). Tumor overgrowth restenosis was confirmed in 17 patients (25.0%) a median of 66 days after stenting (IQR, 34–144). Twelve underwent repeat stenting, two underwent gastric bypass surgery, and three received conservative management because of poor performance status. Stent migration occurred in two patients (2.9%), neither of whom developed restenosis. The cumulative incidence of restenosis by the Aalen–Johansen method was 15.8% at 90 days, 19.1% at 180 days, and 25.0% at 1 year. Sixty-four patients (94.1%) died during follow-up, with a median overall survival of 323 days.

### 3.3. Baseline Inflammatory, Nutritional, and Immune Indices

No demographic, oncological, or procedural variables—including clinical M1 status, metastatic sites, histologic differentiation, Lauren classification, biliary obstruction, and prior chemotherapy—differed significantly between patients with and without restenosis (Table 1), although longer stents (*p* = 0.060) and longer strictures (*p* = 0.084) showed borderline associations. Lymphocyte count was lower, neutrophil count higher, and CRP markedly elevated in patients who developed restenosis (all *p* ≤ 0.034), whereas albumin and platelets did not differ. Five of the six composite indices differed significantly between groups (SII, NLR, CAR, mGPS, and CALLY; all *p* ≤ 0.020); no restenosis occurred in patients with mGPS = 0. CALLY showed the greatest separation, with a median value about seven-fold lower in patients who developed restenosis (0.048 vs. 0.322; *p* < 0.001). PNI did not differ significantly (*p* = 0.141).

### 3.4. Discriminatory Performance of the Composite Indices

**Figure 2:**
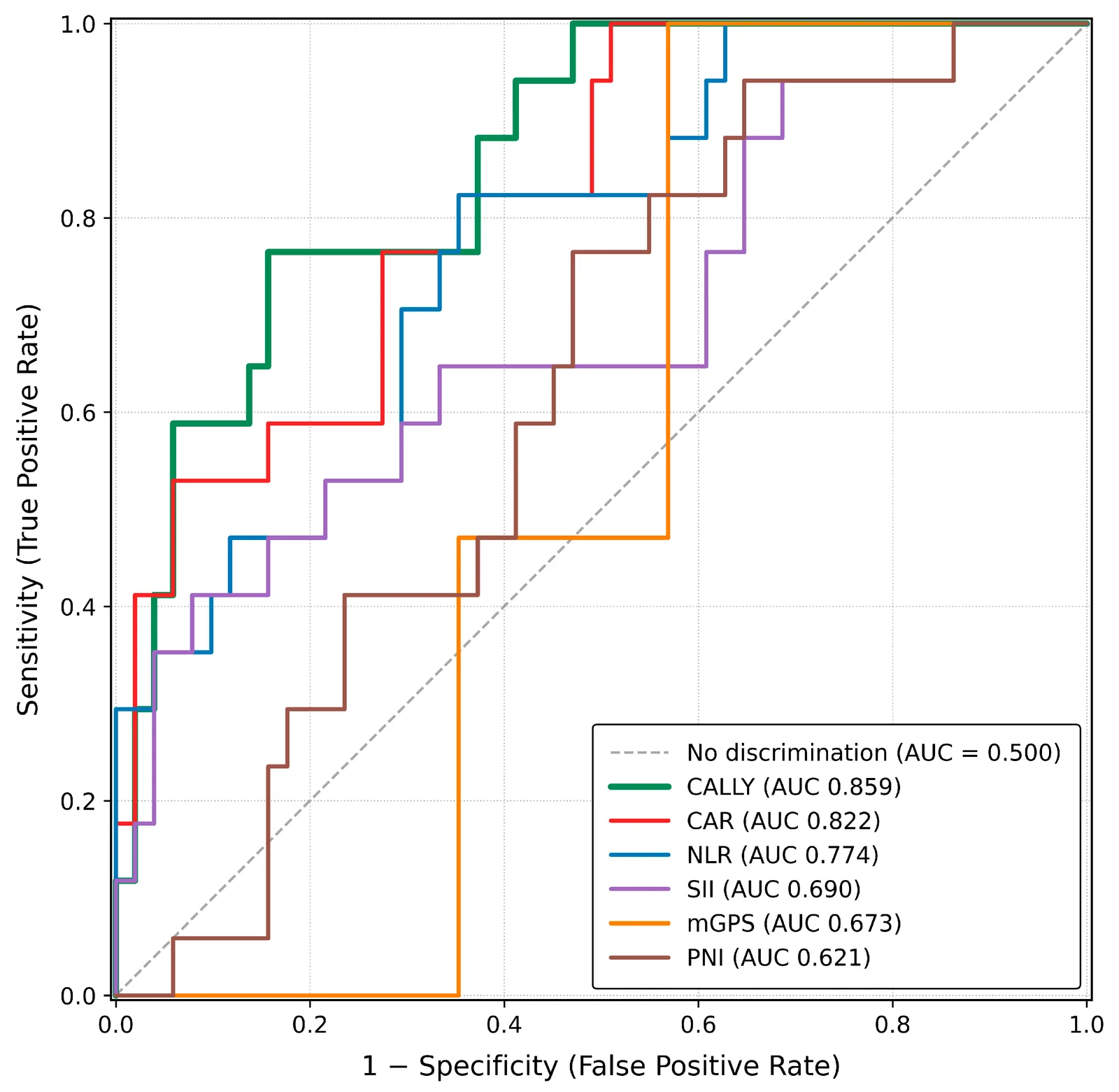
Receiver operating characteristic curves of six preprocedural indices for predicting tumor overgrowth restenosis. The AUC values were 0.859 for CALLY, 0.822 for CAR, 0.774 for NLR, 0.690 for SII, 0.673 for mGPS, and 0.621 for PNI. CALLY (bold green line) had the highest numerical AUC. Pairwise DeLong comparisons with Bonferroni correction showed no significant differences among CALLY, CAR, and NLR. CALLY and CAR had significantly higher AUCs than PNI and mGPS after correction. The diagonal dotted line indicates no discrimination (AUC = 0.500). AUC, area under the receiver operating characteristic curve; CALLY, C-reactive protein–albumin–lymphocyte index; CAR, C-reactive protein-to-albumin ratio; mGPS, modified Glasgow prognostic score; NLR, neutrophil-to-lymphocyte ratio; PNI, prognostic nutritional index; SII, systemic immune-inflammation index.

ROC analysis (Table 2, Figure 2) showed that CALLY had the highest AUC (0.859; 95% CI, 0.758–0.949), followed by CAR (0.822) and NLR (0.774); SII, mGPS, and PNI had lower AUCs. At the exploratory, internally derived cutoff of ≤0.110, CALLY combined a sensitivity of 76.5% with a specificity of 84.3%. Among the continuous indices, CAR had the highest sensitivity (94.1%) but lower specificity, and mGPS ≥ 1 had 100% sensitivity and a 100% negative predictive value, since no patient with mGPS = 0 developed restenosis. Pairwise DeLong comparisons (Table S1) showed no significant differences among CALLY, CAR, and NLR, but both CALLY and CAR exceeded PNI and mGPS after Bonferroni correction. The optimism-corrected AUC for CALLY from 1000 bootstrap iterations was 0.83 (Table S2). Wilson 95% CIs for sensitivity, specificity, and predictive values are provided in Table 2. Time-dependent AUCs accounting for death as a competing risk gave consistent results (CALLY, 0.890 at 90 days and 0.884 at 180 days; CAR, 0.886 and 0.885; Table S10); by this metric, CALLY and CAR discriminated almost identically.

### 3.5. Univariable and Competing-Risk Analyses

**Figure 3:**
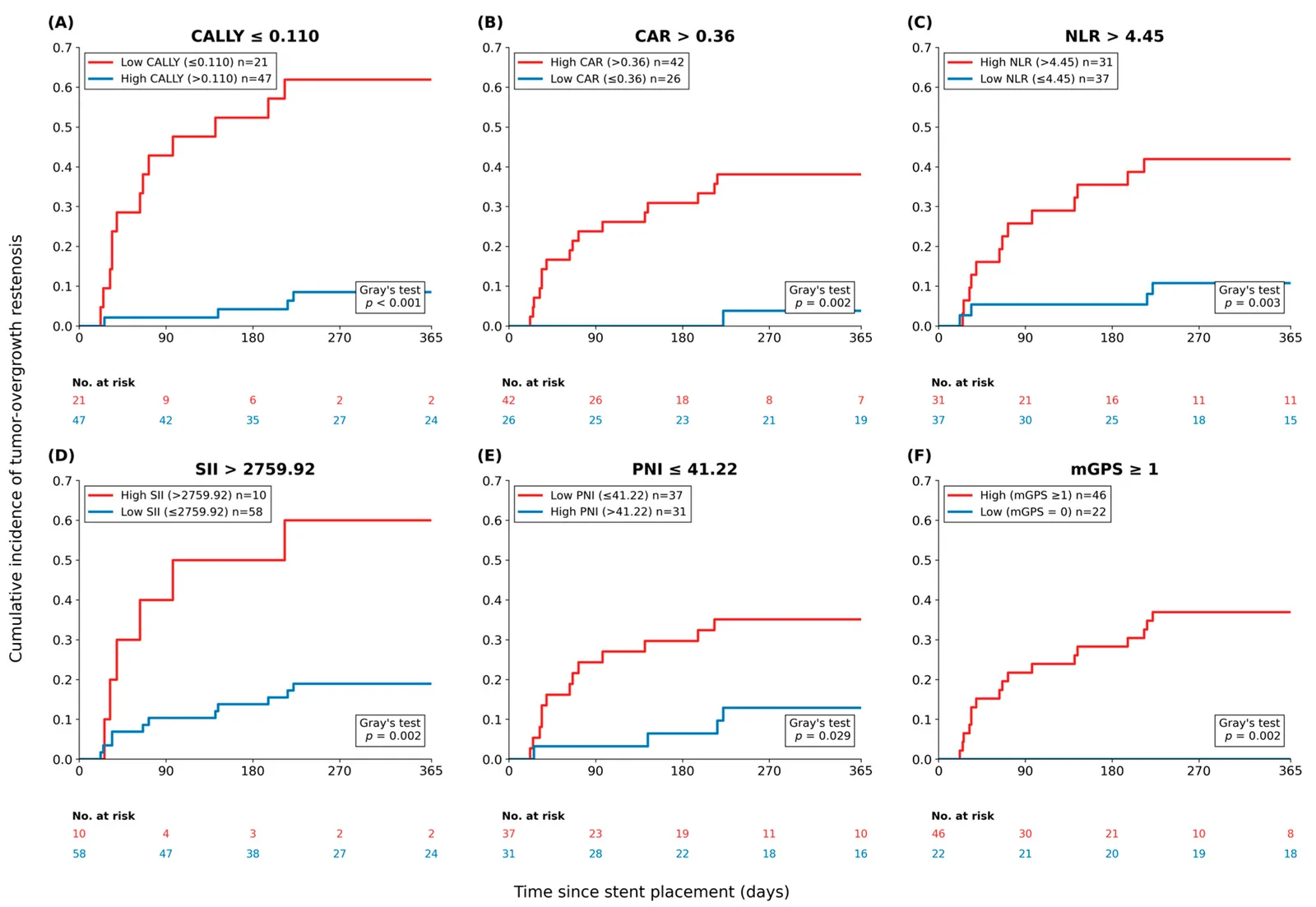
Cumulative incidence of tumor overgrowth restenosis stratified by inflammatory, nutritional, and immune indices. Cumulative incidence function curves for restenosis after partially covered duodenal stenting were estimated using the Aalen–Johansen method, with death without restenosis treated as a competing event. Red denotes the high-risk stratum and blue the low-risk stratum. Exploratory Youden index cutoffs for the continuous indices were (**A**) CALLY ≤ 0.110, (**B**) CAR > 0.36, (**C**) NLR > 4.45, (**D**) SII > 2759.92, and (**E**) PNI ≤ 41.22; for mGPS (**F**), the clinical cutoff of ≥1 was used. *p* values were obtained using Gray’s test. CALLY, C-reactive protein–albumin–lymphocyte; CAR, C-reactive protein-to-albumin ratio; mGPS, modified Glasgow prognostic score; NLR, neutrophil-to-lymphocyte ratio; PNI, prognostic nutritional index; SII, systemic immune-inflammation index. Numbers at risk in each stratum are shown below each panel.

**Figure 4:**
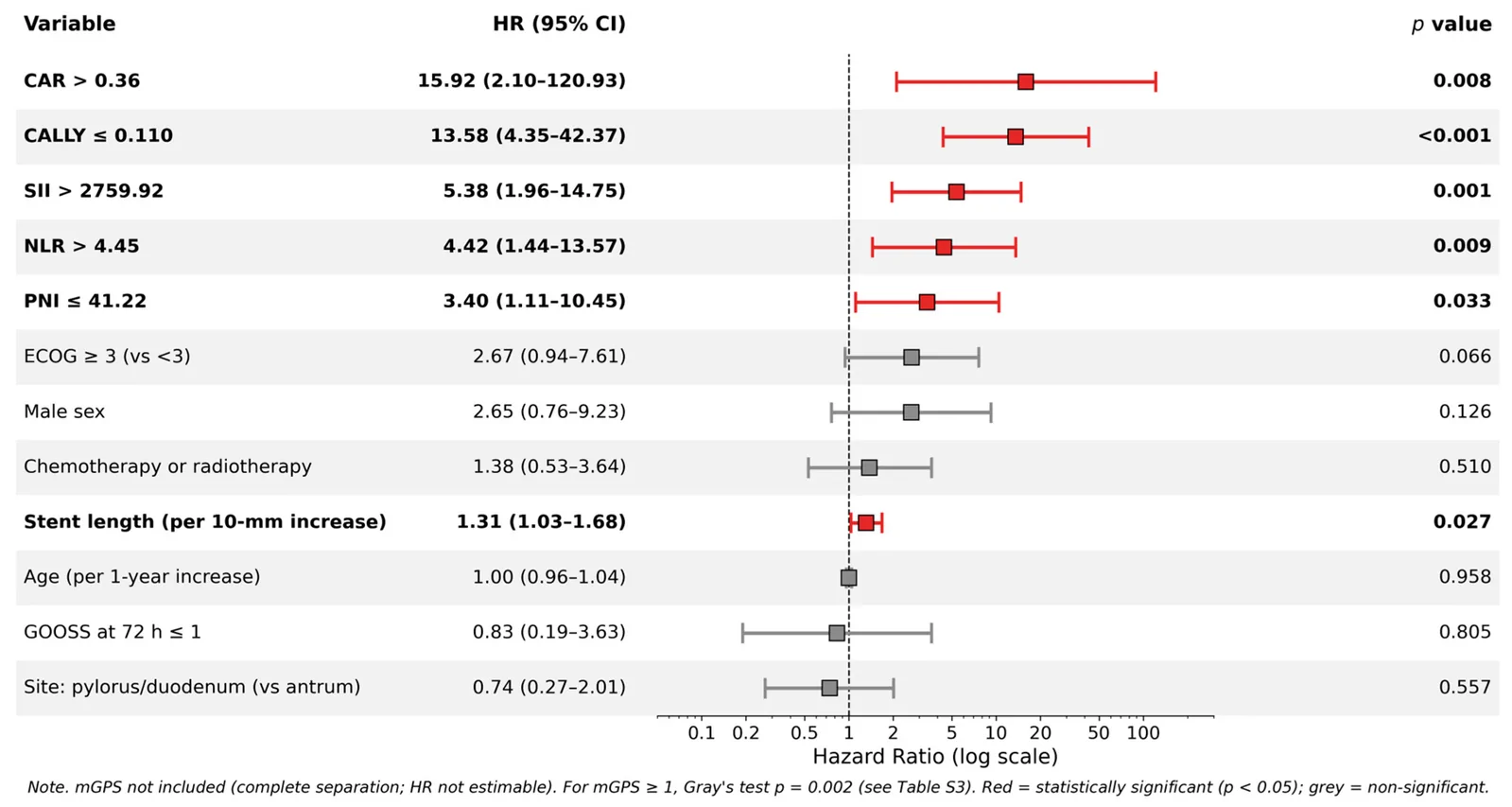
Forest plot of univariable cause-specific Cox proportional hazards analyses for tumor overgrowth restenosis. Hazard ratios and 95% confidence intervals are shown on a logarithmic scale. Red markers indicate *p* < 0.05; gray markers indicate *p* ≥ 0.05. Chemotherapy or radiotherapy denotes post-stenting treatment modeled as a time-fixed covariate. mGPS is not included in the figure because complete separation (0 events in the mGPS = 0 group) prevented reliable hazard ratio estimation; the corresponding Gray’s test *p* value (0.002) is reported in Table S3. CALLY, C-reactive protein–albumin–lymphocyte; CAR, C-reactive protein-to-albumin ratio; ECOG, Eastern Cooperative Oncology Group; mGPS, modified Glasgow prognostic score; NLR, neutrophil-to-lymphocyte ratio; PNI, prognostic nutritional index; SII, systemic immune-inflammation index.

In univariable Cox analysis, ECOG performance status ≥ 3 showed a borderline association with restenosis (*p* = 0.066); stent length was significantly associated (HR per 10 mm increase, 1.31; *p* = 0.027), whereas chemotherapy or radiotherapy was not (*p* = 0.510). All five continuous indices were significantly associated with restenosis when dichotomized at their exploratory cutoffs (Table S3). For mGPS, complete separation prevented reliable HR estimation, but Gray’s test yielded *p* = 0.002. Competing-risk analysis confirmed significant stratification for all six indices (Figure 3); CALLY showed marked separation, with a 180-day cumulative incidence of 52.4% (95% CI, 33.3–71.4) in the low-CALLY group vs. 4.3% (95% CI, 0.0–10.6) in the high-CALLY group (Gray’s test *p* < 0.001). The corresponding univariable hazard ratios are displayed as a forest plot (Figure 4). Competing-risk estimates at 90 and 180 days with bootstrap 95% CIs are summarized in Table S4.

### 3.6. Multivariable Analyses

In the six index-specific primary multivariable models restricted to preprocedural covariates (Table 3), each index with an estimable hazard ratio remained associated with restenosis after adjustment. For mGPS, complete separation again precluded reliable HR estimation, although Gray’s test confirmed significant stratification. The CALLY-based model had the numerically highest C-index (0.836; aHR for CALLY ≤ 0.110, 12.30; 95% CI, 3.89–38.83; *p* < 0.001), followed by the CAR-based (C-index, 0.779) and NLR-based (C-index, 0.738) models. Within the CALLY-based model, stent length was no longer significantly associated with restenosis (aHR per 10 mm increase, 1.20; 95% CI, 0.95–1.51). The CALLY association remained strong when CALLY was analyzed as a continuous variable: each one-standard-deviation increase in log-CALLY was associated with a 91% lower cause-specific hazard of restenosis (adjusted HR per SD, 0.09; 95% CI, 0.03–0.26; C-index, 0.879), with no evidence of nonlinearity (*p* = 0.651; Table S9, Figure S1). The CALLY estimate was essentially unchanged whether post-stenting chemotherapy/radiotherapy was modeled as a time-fixed covariate (aHR, 15.54; 95% CI, 4.85–49.81; Table S6) or as a time-dependent covariate (aHR, 13.59; 95% CI, 4.20–43.98; Table S7); in the latter model, chemotherapy/radiotherapy itself was not associated with restenosis (aHR, 1.93; 95% CI, 0.70–5.30; *p* = 0.202). Adding clinical M1 status, peritoneal metastasis, poorly differentiated or signet-ring cell histology, prior chemotherapy, or ECOG performance status ≥ 3 one at a time did not materially alter the CALLY estimate (aHR range, 11.9–17.1; Table S8), nor did replacing stent length with measured stricture length (aHR, 13.06; 95% CI, 4.15–41.02; C-index, 0.829; Table S8). In a univariable Fine–Gray sensitivity analysis, CALLY ≤ 0.110 was likewise associated with the cumulative incidence of restenosis (subdistribution HR, 5.88; 95% CI, 2.33–14.89; *p* < 0.001; Table S5). The CALLY association was also essentially unchanged when the two patients receiving antibiotics at baseline were excluded (aHR, 16.30; 95% CI, 4.47–59.50; C-index, 0.857; Table S8).

## 4. Discussion

PCDS placement was technically successful in all 68 patients without major procedural complications. Despite this, 17 patients (25%) developed tumor overgrowth restenosis at a median of 66 days. Of the six preprocedural indices examined, CALLY, CAR, and NLR showed the highest numerical discrimination, and CALLY—the only index combining all three host domains—had the highest numerical AUC and C-index (0.836; aHR, 12.30 for CALLY ≤ 0.110 in the primary model restricted to preprocedural covariates), although pairwise differences among CALLY, CAR, and NLR were not statistically significant. Given the small number of events, the magnitude of this hazard ratio is imprecise and potentially unstable and requires confirmation in larger cohorts.

The procedural pattern matches what the partially covered large-flare design was developed to achieve: migration occurred in only 2.9% of patients, and no ingrowth was observed. Tumor overgrowth was the dominant mode of late failure and occurred early—at a median of 66 days, almost always before death. Stent geometry alone, therefore, does not prevent this complication. Salvage options after restenosis were limited: 12 patients required repeat stenting, 2 underwent gastric bypass, and 3 received conservative care only. Identifying patients at high preprocedural risk could therefore be clinically relevant if a reliable risk marker is validated.

The observed association suggests that systemic host status may contribute to the risk of tumor overgrowth at the uncovered stent ends, in addition to tumor-related factors. CALLY, CAR, and NLR yielded the highest AUCs without significant differences among them, while CALLY and CAR outperformed PNI and mGPS after Bonferroni correction. This pattern suggests that indices incorporating CRP may capture inflammatory features relevant to restenosis, although the present study cannot determine which individual component drives the association. CALLY partially overlaps with the other leading indices: it shares CRP and albumin with CAR and lymphocyte count with NLR. These overlapping components may partly explain their correlated and similar discriminatory performance, although the indices are not interchangeable. CALLY had the numerically highest discrimination; however, pairwise differences versus CAR and NLR were not statistically significant. CALLY incorporates all three host domains in a single value and shows the most balanced sensitivity and specificity. Because its components are part of routine preprocedural laboratory testing, CALLY is readily obtainable; if externally validated, it could be evaluated in prospective studies as a risk-stratification marker to test whether intensified surveillance or alternative management strategies, such as EUS-guided gastrojejunostomy, improve clinical outcomes. Throughout, the 0.110 threshold should be regarded as an exploratory, internally derived cutoff. The consistent continuous-variable result (adjusted HR per one-standard-deviation increase in log-CALLY, 0.09; 95% CI, 0.03–0.26) avoids reliance on data-driven dichotomization and supports the consistency of the observed association. Because this estimate is expressed on the log-transformed scale, it should be interpreted as an association rather than a causal or absolute-risk effect. External validation is required before clinical implementation.

Several biological mechanisms could plausibly connect a heightened preprocedural inflammatory state with tumor overgrowth, although none were assessed directly in this study and all remain hypothetical. CRP is an acute-phase reactant downstream of interleukin-6 signaling, which has been linked in preclinical studies to tumor proliferation, angiogenesis, and extracellular-matrix remodeling [25]. Relative lymphopenia may reflect impaired antitumor immunosurveillance, whereas hypoalbuminemia may reflect inflammation-associated catabolism and poor nutritional status. Whether reactions at the tumor–stent interface amplify these processes is unknown. Correlative studies measuring circulating cytokines, together with histologic examination of overgrown tissue, will be needed before any mechanistic interpretation can be made.

Post-stenting chemotherapy or radiotherapy warrants separate comment. By design, the primary prediction models included only information available before stenting, and post-stenting treatment was evaluated solely in sensitivity analyses. Because it begins after baseline, modeling it as a fixed covariate invites immortal-time and treatment-selection bias, and it was indeed significantly associated with restenosis in the time-fixed mGPS-based model and showed a borderline association in the CAR-based model (*p* = 0.048 and 0.051, respectively; Table S6). Once modeled as a time-dependent covariate, however, the association disappeared (aHR, 1.93; 95% CI, 0.70–5.30; *p* = 0.202; Table S7), while the CALLY association was unaffected. A plausible explanation is treatment selection and differential survival: patients selected for post-stenting oncologic therapy may have had better performance status and longer survival, providing more opportunity for restenosis to be observed. The finding does not imply that therapy itself is harmful.

NLR, SII, PNI, mGPS, and CAR have been linked to overall survival, postoperative outcomes, and chemotherapy tolerance after radical gastrectomy or palliative systemic therapy [22,23,24,26,27,28]. Their prognostic value in palliative enteral stenting has rarely been examined [29]. CRP-based indices, including CALLY, have not previously been evaluated as predictors of stent failure. The present study therefore identifies a new application for these widely available indices in patients receiving palliative stenting for malignant GOO.

These findings should also be considered alongside established clinical predictors and emerging procedural alternatives. Prior multicenter data identified poor performance status, longer or multifocal stenoses, biliary obstruction, and liver metastasis as predictors of clinical failure, stent dysfunction, or tumor overgrowth; an international registry reported stent ingrowth or overgrowth in 12.4% of patients; and a randomized trial found fewer 6-month reinterventions with EUS-guided gastroenterostomy than with duodenal stenting [30,31,32]. Consistent with the view that stent length mainly reflects local tumor extent, measured stricture length correlated strongly with stent length in our cohort (Spearman ρ = 0.93), and neither remained significantly associated with restenosis once CALLY was taken into account.

Two methodological features strengthen our analysis. First, data were drawn from a prospectively maintained cohort database, with baseline laboratory and procedural details recorded in real time. Second, because all 68 patients received the same partially covered stent (HANAROSTENT Kim’s Flare), heterogeneity attributable to stent design was minimized.

This study has several limitations. First, it was conducted at a single center with a modest sample size and only 17 restenosis events; although the primary models were deliberately limited to three parameters, the resulting events-per-parameter ratio (approximately 5.7) falls short of conventional recommendations, so the large hazard ratios with wide CIs should be read as exploratory estimates of association rather than precise effect sizes. Second, the indices were built from overlapping laboratory variables and were strongly correlated, and the exploratory CALLY cutoff of ≤0.110 was derived from this same cohort using the Youden index; despite bootstrap correction of the apparent AUC for optimism and consistent continuous-variable results, the cutoff is not ready for clinical use and needs external validation in larger, independent, multicenter cohorts. Third, mortality was high and constituted a considerable competing risk; the Aalen–Johansen, Fine–Gray, and time-dependent analyses address this, but residual confounding cannot be excluded. Fourth, because all patients had unresectable disease, staging was limited to the clinical M category, and tumor burden could not be characterized beyond metastatic sites and stricture length, leaving room for residual confounding by unmeasured tumor factors. Fifth, although patients were followed at predefined intervals, confirmatory endoscopy or imaging was symptom-triggered, so restenosis may have been detected preferentially in patients who survived longer or were followed more closely. Finally, two patients were receiving antibiotics for mild bronchopneumonia at baseline sampling, which may have influenced the CRP-based indices; the CALLY association was essentially unchanged in a sensitivity analysis excluding these two patients (aHR, 16.30; 95% CI, 4.47–59.50).

## 5. Conclusions

Partially covered duodenal stenting was technically successful in every patient, yet one in four developed tumor overgrowth restenosis at a median of 66 days. Among the six preprocedural indices, the C-reactive protein-based indices showed the highest numerical discrimination for subsequent restenosis, with no statistically significant pairwise differences among CALLY, CAR, and NLR, and the association held across continuous, competing-risk, time-dependent, and tumor-covariate-adjusted analyses. These findings support CALLY as a promising exploratory biomarker for risk stratification, not as a validated clinical decision tool. Whether CALLY-guided surveillance or the selection of alternatives such as EUS-guided gastroenterostomy improves patient outcomes remains a hypothesis for prospective study, and the proposed cutoff requires external validation in larger, independent, multicenter cohorts before clinical implementation.

## Figures and Tables

**Table 1:** Clinical characteristics of the study cohort according to subsequent tumor overgrowth restenosis.

Variable	Total (*n* = 68)	No Restenosis (*n* = 51)	Restenosis (*n* = 17)	*p*-Value
Age (years), median (IQR)	60.0 (53.0–69.0)	60.0 (53.0–69.0)	59.0 (50.0–69.0)	0.671
Male, *n* (%)	46 (67.6)	32 (62.7)	14 (82.4)	0.230
ECOG performance, *n* (%)				0.215
1	35 (51.5)	29 (56.9)	6 (35.3)	
2	21 (30.9)	15 (29.4)	6 (35.3)	
≥3	12 (17.6)	7 (13.7)	5 (29.4)	
Site of stenosis, *n* (%)				>0.999
Antrum	25 (36.8)	19 (37.3)	6 (35.3)	
Pylorus or duodenum	43 (63.2)	32 (62.7)	11 (64.7)	
Baseline GOOSS, *n* (%)				0.737
0	14 (20.6)	10 (19.6)	4 (23.5)	
1	54 (79.4)	41 (80.4)	13 (76.5)	
72 h GOOSS, median (IQR)	2.0 (2.0–2.0)	2.0 (2.0–2.0)	2.0 (2.0–2.0)	0.170
Stent length (mm), median (IQR)	100 (80–120)	100 (80–120)	120 (80–120)	0.060
Post-stenting chemotherapy or radiotherapy, *n* (%)	32 (47.1)	22 (43.1)	10 (58.8)	0.279
Prior chemotherapy before stenting, *n* (%)	24 (35.3)	16 (31.4)	8 (47.1)	0.257
Distant metastasis (clinical M1), *n* (%)	23 (33.8)	16 (31.4)	7 (41.2)	0.557
Liver metastasis, *n* (%)	5 (7.4)	3 (5.9)	2 (11.8)	0.592
Peritoneal metastasis, *n* (%)	10 (14.7)	8 (15.7)	2 (11.8)	>0.999
Distant lymph node stations, *n* (%)	7 (10.3)	5 (9.8)	2 (11.8)	>0.999
Biliary obstruction, *n* (%)	3 (4.4)	1 (2.0)	2 (11.8)	0.152
Histologic differentiation, *n* (%)				0.223
WD	7 (10.3)	7 (13.7)	0 (0.0)	
MD	22 (32.4)	15 (29.4)	7 (41.2)	
PD	22 (32.4)	18 (35.3)	4 (23.5)	
SRC	17 (25.0)	11 (21.6)	6 (35.3)	
Lauren classification, *n* (%)				0.751
Intestinal	22 (32.4)	17 (33.3)	5 (29.4)	
Diffuse	35 (51.5)	25 (49.0)	10 (58.8)	
Mixed	11 (16.2)	9 (17.6)	2 (11.8)	
Stricture length (cm), median (IQR)	6.5 (5.0–8.2)	6.0 (5.0–8.0)	8.0 (6.0–10.0)	0.084
Clinical success, *n* (%)	64 (94.1)	47 (92.2)	17 (100.0)	>0.999
Laboratory values, median (IQR)				
WBC, ×10^9^/L	8.04 (5.98–9.95)	7.56 (5.54–9.41)	9.19 (7.43–11.55)	0.074
ANC, /mm^3^	5860 (3511–7661)	5060 (3121–6968)	6736 (5052–10,109)	0.034
ALC, /mm^3^	1256 (915–1671)	1476 (984–1817)	973 (799–1140)	0.004
Platelet, ×10^9^/L	262.5 (210.0–337.8)	267.0 (213.5–342.5)	247.0 (171.0–303.0)	0.470
Albumin, g/dL	3.5 (2.9–3.7)	3.5 (3.0–3.8)	3.5 (2.9–3.7)	0.777
CRP, mg/dL	1.87 (0.82–3.89)	1.42 (0.57–2.97)	5.60 (2.77–9.57)	<0.001
Inflammatory, nutritional, and immunological indices, median (IQR)				
SII	1011.6 (629.4–2219.6)	964.9 (534.3–1550.0)	1928.9 (777.6–3447.5)	0.020
NLR	4.08 (2.88–7.26)	3.78 (2.67–6.43)	7.07 (4.72–11.79)	<0.001
CAR	0.57 (0.22–1.20)	0.38 (0.14–0.96)	1.93 (0.77–3.17)	<0.001
PNI	40.4 (34.9–45.0)	42.3 (35.4–48.4)	38.9 (33.7–41.2)	0.141
CALLY	0.184 (0.088–0.599)	0.322 (0.138–0.948)	0.048 (0.034–0.110)	<0.001
mGPS, *n* (%)				0.002
0	22 (32.4)	22 (43.1)	0 (0.0)	
1	20 (29.4)	11 (21.6)	9 (52.9)	
2	26 (38.2)	18 (35.3)	8 (47.1)	
Follow-up period (days), median (IQR)	347 (146–566)	371 (167–573)	262 (88–415)	—
Death during follow-up, *n* (%)	64 (94.1)	47 (92.2)	17 (100.0)	0.565
Stent migration, *n* (%)	2 (2.9)	2 (3.9)	0 (0.0)	>0.999

Continuous variables are presented as median (interquartile range, IQR) and were compared using the Wilcoxon rank-sum test. Categorical variables are presented as *n* (%) and were compared using Fisher’s exact test or the chi-square test, as appropriate. CALLY was calculated as (albumin [g/dL] × ALC [cells/µL])/(CRP [mg/dL] × 10^4^). Abbreviations: ALC, absolute lymphocyte count; ANC, absolute neutrophil count; CALLY, C-reactive protein–albumin–lymphocyte; CAR, C-reactive protein-to-albumin ratio; CRP, C-reactive protein; ECOG, Eastern Cooperative Oncology Group; GOOSS, Gastric Outlet Obstruction Scoring System; IQR, interquartile range; mGPS, modified Glasgow Prognostic Score; NLR, neutrophil-to-lymphocyte ratio; PNI, prognostic nutritional index; SII, systemic immune-inflammation index; WBC, white blood cell count. Clinical M staging only was available because no patient underwent surgical resection. WD/MD/PD, well/moderately/poorly differentiated; SRC, signet-ring cell carcinoma. —, not applicable.

**Table 2:** Discriminatory performance of inflammatory, nutritional, and immunological indices for predicting tumor overgrowth restenosis.

Index	AUC	95% CI (DeLong)	Cutoff Used	Sensitivity, % (95% CI)	Specificity, % (95% CI)	PPV/NPV, % (95% CI)
CALLY	0.859	0.758–0.949	≤0.110	76.5 (52.7–90.4)	84.3 (72.0–91.8)	61.9 (40.9–79.2)/91.5 (80.1–96.6)
CAR	0.822	0.698–0.927	>0.36	94.1 (73.0–99.0)	49.0 (35.9–62.3)	38.1 (25.0–53.2)/96.2 (81.1–99.3)
NLR	0.774	0.646–0.887	>4.45	76.5 (52.7–90.4)	64.7 (51.0–76.4)	41.9 (26.4–59.2)/89.2 (75.3–95.7)
SII	0.690	0.531–0.835	>2759.92	35.3 (17.3–58.7)	92.2 (81.5–96.9)	60.0 (31.3–83.2)/81.0 (69.1–89.1)
mGPS	0.673	0.555–0.783	≥1	100.0 (81.6–100.0)	43.1 (30.5–56.7)	37.0 (24.5–51.4)/100.0 (85.1–100.0)
PNI	0.621	0.478–0.766	≤41.22	76.5 (52.7–90.4)	52.9 (39.5–65.9)	35.1 (21.8–51.2)/87.1 (71.1–94.9)

Exploratory, internally derived cutoff values for the continuous indices were determined using the Youden index in receiver-operating characteristic (ROC) curve analysis; for mGPS, the clinical cutoff of 0 vs. ≥1 was used. The 95% confidence intervals for AUCs were calculated using the DeLong method.

**Table 3:** Adjusted time-to-event analyses for tumor overgrowth restenosis.

Model	Variable	aHR	95% CI	*p*-Value
Model 1 (CALLY)	CALLY ≤ 0.110	12.30	3.89–38.83	<0.001
Model 1 (CALLY)	Pylorus/duodenum (vs. antrum)	0.90	0.33–2.47	0.838
Model 1 (CALLY)	Stent length (per 10 mm increase)	1.20	0.95–1.51	0.132
Model 1 (CALLY)	C-index = 0.836			
Model 2 (CAR)	CAR > 0.36	15.47	2.02–118.23	0.008
Model 2 (CAR)	Pylorus/duodenum (vs. antrum)	1.18	0.43–3.25	0.756
Model 2 (CAR)	Stent length (per 10 mm increase)	1.31	1.00–1.70	0.046
Model 2 (CAR)	C-index = 0.779			
Model 3 (NLR)	NLR > 4.45	4.47	1.45–13.77	0.009
Model 3 (NLR)	Pylorus/duodenum (vs. antrum)	0.86	0.31–2.36	0.769
Model 3 (NLR)	Stent length (per 10 mm increase)	1.33	1.03–1.70	0.027
Model 3 (NLR)	C-index = 0.738			
Model 4 (SII)	SII > 2759.92	5.11	1.68–15.55	0.004
Model 4 (SII)	Pylorus/duodenum (vs. antrum)	0.60	0.20–1.79	0.359
Model 4 (SII)	Stent length (per 10 mm increase)	1.21	0.93–1.57	0.150
Model 4 (SII)	C-index = 0.734			
Model 5 (PNI)	PNI ≤ 41.22	3.37	1.09–10.37	0.034
Model 5 (PNI)	Pylorus/duodenum (vs. antrum)	0.97	0.35–2.67	0.952
Model 5 (PNI)	Stent length (per 10 mm increase)	1.32	1.03–1.71	0.031
Model 5 (PNI)	C-index = 0.718			
Model 6 (mGPS)	mGPS ≥ 1	—	—	—*
Model 6 (mGPS)	Pylorus/duodenum (vs. antrum)	1.02	0.37–2.84	0.965
Model 6 (mGPS)	Stent length (per 10 mm increase)	1.32	1.03–1.71	0.032
Model 6 (mGPS)	C-index not reported (complete separation)			

Because the six inflammatory, nutritional, and immunological indices were strongly correlated, each index was entered into a separate cause-specific Cox proportional hazards model together with the preprocedural covariates of stenosis site and stent length; post-stenting chemotherapy/radiotherapy was examined separately in sensitivity analyses (Tables S6 and S7). Models are presented in descending order of C-index. * For mGPS ≥ 1, the Cox aHR could not be reliably estimated because of complete separation (0 events in the mGPS = 0 group); the adjusted Cox effect could not be estimated; in the unadjusted competing-risk analysis, Gray’s test *p* = 0.002 (Figure 3). The C-index is not reported. Cutoffs for the continuous indices are exploratory, internally derived values (Youden index) and require external validation; mGPS was categorized using the clinical cutoff of 0 vs. ≥1. Abbreviations: aHR, adjusted hazard ratio; CI, confidence interval.

## Data Availability

The data presented in this study are available on request from the corresponding author due to privacy restrictions relating to participant confidentiality. The data are not publicly available because they contain information that could compromise the privacy of research participants.

## Supplementary Materials

The following supporting information can be downloaded at https://www.mdpi.com/article/10.3390/cancers18172803/s1: Table S1: Pairwise differences in AUCs and Bonferroni-adjusted *p* values; Table S2: Bootstrap-based optimism correction of the CALLY index AUC; Table S3: Univariable Cox proportional hazards analysis for time to tumor overgrowth restenosis; Table S4: Cumulative incidence of tumor overgrowth restenosis at 90 and 180 days after stent placement with bootstrap 95% confidence intervals; Table S5: Univariable Fine-Gray subdistribution hazard ratio for CALLY ≤ 0.110; Table S6: Cause-specific Cox models with post-stenting chemotherapy/radiotherapy as a time-fixed covariate; Table S7: Cause-specific Cox model for tumor overgrowth restenosis with post-stenting chemotherapy/radiotherapy as a time-dependent covariate; Table S8: Sensitivity analyses assessing the stability of the CALLY association; Table S9: Continuous-variable cause-specific Cox models (per one standard deviation) adjusted for stenosis site and stent length; Table S10: Time-dependent AUCs for tumor overgrowth restenosis at 90 and 180 days; Table S11: STROBE statement—checklist of items that should be included in reports of cohort studies; Figure S1: Restricted cubic spline analysis of continuous CALLY; Figure S2: Patient selection flowchart.
