# Supplementary material for: C-Reactive Protein-Based Composite Indices for Predicting Tumor Overgrowth Restenosis After Partially Covered Duodenal Stenting in Gastric Cancer: A Cohort Study
Source: Cancers (Basel). 2026 Aug 28;18(17):2803. doi: 10.3390/cancers18172803 (PMC13565600; doi:10.3390/cancers18172803)
Supplement: Supplementary file 1 [file cancers-18-02803-s001.zip › cancers-4497872-supplementary.pdf]

Supplementary Materials

C-Reactive Protein-Based Composite Indices for Predicting Tumor Overgrowth Restenosis after Partially Covered Duodenal Stenting in Gastric Cancer: A Cohort Study

Hyuk Lee, Young Eun Oh, Tae-Se Kim, Yang Won Min, Byung-Hoon Min and Jun Haeng Lee

S1. Pairwise comparisons of AUCs (DeLong method with Bonferroni correction)

Pairwise comparisons of the six ROC curves were performed using the DeLong method for correlated AUCs, implemented in SAS PROC LOGISTIC (ROC and ROCCONTRAST statements). Bonferroni correction was applied for the 15 pairwise comparisons. AUC differences are shown in the cells (row variable − column variable; positive values indicate that the row variable had a higher AUC). Statistical significance is indicated based on Bonferroni-corrected p values: * $p < 0.05$; ** $p < 0.01$; *** $p < 0.001$. No symbol indicates $p \geq 0.05$ after correction. The three indices with the highest AUCs (CALLY, CAR, and NLR) did not differ significantly from one another after correction, whereas the two CRP-based indices (CALLY and CAR) showed significantly greater discrimination than PNI and mGPS.

Table S1. Pairwise differences in AUCs and Bonferroni-adjusted p values

	CALLY	CAR	NLR	SII	mGPS	PNI
**CALLY**	—	+0.037	+0.085	+0.170	+0.186**	+0.239***
**CAR**		—	+0.048	+0.133	+0.149*	+0.202*
**NLR**			—	+0.084	+0.101	+0.153
**SII**				—	+0.017	+0.069
**mGPS**					—	+0.052
**PNI**						—

The cells show differences in AUC values from pairwise comparisons using the DeLong method (row variable − column variable; positive values indicate that the row variable had a higher AUC). Statistical significance is based on Bonferroni-corrected p values for 15 comparisons: * $p < 0.05$; ** $p < 0.01$; *** $p < 0.001$. No symbol indicates $p \geq 0.05$ after correction. The three indices with the highest AUCs (CALLY, CAR, and NLR) did not differ significantly from one another; however, the two CRP-based indices (CALLY and CAR) showed significantly higher AUCs than PNI and mGPS.

Abbreviations: AUC, area under the receiver operating characteristic curve; CALLY, C-reactive protein–albumin–lymphocyte; CAR, C-reactive protein-to-albumin ratio; CI, confidence interval; mGPS, modified Glasgow Prognostic Score; NLR, neutrophil-to-lymphocyte ratio; NPV, negative predictive value; PNI, prognostic nutritional index; PPV, positive predictive value; SII, systemic immune-inflammation index.

Table S1 (continued). Raw and Bonferroni-adjusted p values for all pairwise AUC comparisons

Comparison	AUC difference	Raw p	Bonferroni-adjusted p
CALLY vs. CAR	0.037	0.247	1.000
CALLY vs. NLR	0.085	0.205	1.000

Comparison	AUC difference	Raw p	Bonferroni-adjusted p
CALLY vs. SII	0.170	0.037	0.553
CALLY vs. mGPS	0.186	<0.001	0.003
CALLY vs. PNI	0.239	<0.001	<0.001
CAR vs. NLR	0.048	0.549	1.000
CAR vs. SII	0.133	0.155	1.000
CAR vs. mGPS	0.149	0.003	0.044
CAR vs. PNI	0.202	0.001	0.022
NLR vs. SII	0.084	0.045	0.671
NLR vs. mGPS	0.101	0.205	1.000
NLR vs. PNI	0.153	0.057	0.855
SII vs. mGPS	0.017	0.855	1.000
SII vs. PNI	0.069	0.432	1.000
mGPS vs. PNI	0.052	0.357	1.000

Pairwise comparisons of AUCs were performed using the DeLong method for correlated ROC curves. Raw p values were Bonferroni-adjusted for the 15 pairwise comparisons (multiplied by 15 and capped at 1.000). The two CRP-based indices (CALLY and CAR) showed significantly higher AUCs than PNI and mGPS after correction (adjusted $p < 0.001$ to $p = 0.044$), whereas the three highest-ranked indices (CALLY, CAR, and NLR) did not differ significantly from one another (all adjusted $p = 1.000$).

S2. Bootstrap-based optimism correction of the CALLY AUC (0.632+ method)

The exploratory CALLY cutoff (≤0.110) was derived using the Youden index in the same cohort, raising the possibility that the apparent AUC could be influenced by optimism bias. To assess this, Efron–Tibshirani 0.632+ correction was applied using a user-defined bootstrap macro with 1,000 resampling iterations (SAS PROC SURVEYSELECT, METHOD=URS). For each bootstrap resample drawn with replacement, the AUC was calculated both within the bootstrap sample and in the observations not selected for that resample (the out-of-bag set). Optimism was estimated as the mean difference between the in-sample (bootstrap) and out-of-bag AUCs, and the optimism-corrected AUC was obtained using the Efron–Tibshirani 0.632+ weighting.

Table S2. Bootstrap-based optimism correction of the CALLY index AUC

Quantity	Value
Apparent AUC (training set)	0.859
Mean bootstrap AUC (B = 1000)	0.857
Mean out-of-bag AUC	0.821
Optimism estimate	0.036
Optimism-corrected AUC (0.632+ method)	0.830

The optimism-corrected AUC (0.83) was very similar to the apparent AUC (0.859), suggesting that the discriminatory performance of CALLY in this cohort was unlikely to be driven primarily by overfitting. Nevertheless, both the cutoff value and overall discriminatory performance should be validated in an independent external cohort before routine clinical implementation. Abbreviations: AUC, area under the curve; CALLY, C-reactive protein–albumin–lymphocyte.

S3. Univariable Cox proportional hazards analysis

Univariable cause-specific Cox proportional hazards regression was performed (SAS PROC PHREG), with death without restenosis treated as a censoring event. Demographic, oncologic, and procedural variables, together with the six inflammation, nutrition, and immune indices categorized using the cutoffs specified in Methods 2.5 of the main manuscript, were each examined separately. For mGPS, the hazard ratio could not be reliably estimated due to complete separation; Gray's test was used instead. Stent length and all five indices with estimable Cox hazard ratios were significantly associated with restenosis; mGPS also significantly stratified cumulative incidence by Gray's test.

Table S3. Univariable Cox proportional hazards analysis for time to tumor overgrowth restenosis

Variable	HR	95% CI	*p*-value
Age (per 1-yr increase)	1.00	0.96–1.04	0.958
Male	2.65	0.76–9.23	0.126
ECOG≥3 (*vs.* <3)	2.67	0.94–7.61	0.066
Site: Gastric outlet/duodenum (*vs.* antrum)	0.74	0.27–2.01	0.557
Post-stenting chemotherapy/radiotherapy (time-fixed)	1.38	0.53–3.64	0.510
Stent length (per 10 mm increase)	1.31	1.03–1.68	0.027
72 h GOOSS≤1	0.83	0.19–3.63	0.805
Clinical success	0.86	0.11–6.54	0.886
CALLY≤0.110	13.58	4.35–42.37	<0.001
CAR>0.36	15.92	2.10–120.93	0.008
NLR>4.45	4.42	1.44–13.57	0.009
SII>2759.92	5.38	1.96–14.75	0.001
PNI≤41.22	3.40	1.11–10.45	0.033
mGPS≥1	—	—	0.002*

For mGPS≥1, the Cox hazard ratio could not be reliably estimated because of complete separation (0 events in the mGPS=0 group). The p value was derived from the Gray's test. Rows shown in bold indicate $p < 0.05$.

Abbreviations: CALLY, C-reactive protein–albumin–lymphocyte; CAR, C-reactive protein-to-albumin ratio; CI, confidence interval; ECOG, Eastern Cooperative Oncology Group; GOOSS, Gastric Outlet Obstruction Scoring System; HR, hazard ratio; mGPS, modified Glasgow Prognostic Score; NLR, neutrophil-to-lymphocyte ratio; PNI, prognostic nutritional index; SII, systemic immune-inflammation index.

S4. Cumulative incidence of restenosis with death as a competing event

Because most patients died during follow-up before restenosis could occur, death represented a substantial competing event. The cumulative incidence function (CIF) of restenosis at 90 and 180 days was estimated using the Aalen–Johansen method (SAS PROC LIFETEST with the EVENTCODE option), with death without restenosis treated as a competing event. These time points capture early and intermediate-term clinical decision-making windows.

Table S4. Cumulative incidence of tumor overgrowth restenosis at 90 and 180 days after stent placement with bootstrap 95% confidence intervals

Index	Stratum	n	Events	Death without restenosis	90-day CIF, % (95% CI)	180-day CIF, % (95% CI)	Gray's test p
CALLY≤0.110	Low CALLY (≤0.110)	21	13	8	42.9 (23.8–61.9)	52.4 (33.3–71.4)	<0.001
CALLY≤0.110	High CALLY (>0.110)	47	4	39	2.1 (0.0–6.4)	4.3 (0.0–10.6)	
CAR>0.36	High	42	16	26	23.8 (11.9–35.7)	31.0 (19.0–45.2)	0.002
CAR>0.36	Low	26	1	21	0.0 (0.0–0.0)	0.0 (0.0–0.0)	
NLR>4.45	High	31	13	18	25.8 (9.7–41.9)	35.5 (19.4–51.6)	0.003
NLR>4.45	Low	37	4	29	5.4 (0.0–13.5)	5.4 (0.0–13.5)	
SII>2759.92	High	10	6	4	40.0 (10.0–70.0)	50.0 (20.0–80.0)	0.002
SII>2759.92	Low	58	11	43	10.3 (3.4–19.0)	13.8 (5.2–22.4)	
PNI≤41.22	Low (PNI≤41.22)	37	13	23	24.3 (10.8–40.5)	29.7 (18.9–44.7)	0.029
PNI≤41.22	High (PNI>41.22)	31	4	24	3.2 (0.0–9.7)	6.5 (0.0–16.1)	
mGPS≥1	High (mGPS≥1)	46	17	29	21.7 (8.7–34.8)	28.3 (15.2–41.3)	0.002
mGPS≥1	Low (mGPS=0)	22	0	18	0.0 (0.0–0.0)	0.0 (0.0–0.0)	

The CIF for restenosis was estimated using the Aalen–Johansen method, with death without restenosis treated as a competing event. Equality of the cumulative incidence functions between strata was compared using Gray's test; the corresponding p values are shown in the last column. Point estimates were recomputed with the Aalen–Johansen estimator and are accompanied by 95% confidence intervals from 2,000 bootstrap resamples; minor differences

from previously reported values reflect this recomputation.

Abbreviations: CALLY, C-reactive protein–albumin–lymphocyte; CAR, C-reactive protein-to-albumin ratio; CIF, cumulative incidence function; mGPS, modified Glasgow Prognostic Score; NLR, neutrophil-to-lymphocyte ratio; PNI, prognostic nutritional index; SII, systemic immune-inflammation index.

S5. Fine–Gray subdistribution hazard sensitivity analysis

In our cohort, 64 patients (94.1%) died during follow-up, representing a substantial competing risk for tumor overgrowth restenosis. In the primary analysis, cause-specific Cox proportional hazards regression was used. Within this framework, death without restenosis was treated as a censoring event, and cumulative incidence functions were estimated using the Aalen–Johansen method.

As a sensitivity analysis, the EVENTCODE= option in SAS PROC PHREG (Fine and Gray, 1999) was used to fit a Fine–Gray subdistribution hazards model for CALLY, dichotomized using the exploratory cutoff value of ≤0.110. This approach yields the subdistribution hazard ratio (sHR), which directly reflects differences in the cumulative incidence of restenosis in the presence of a competing risk.

Table S5. Univariable Fine–Gray subdistribution hazard ratio for CALLY ≤0.110

Index (cut-off)	sHR	95% CI	*p*-value
CALLY ≤ 0.110	5.88	2.33–14.89	<0.001

The univariable Fine–Gray analysis also demonstrated a significant association between low CALLY and the cumulative incidence of tumor overgrowth restenosis (sHR, 5.88; 95% CI, 2.33–14.89; $p < 0.001$). Cause-specific hazard ratios reflect the instantaneous risk of restenosis among patients who remain event-free, whereas subdistribution hazard ratios account for the competing effect of death when estimating cumulative incidence; because the two approaches estimate different quantities—and the Fine–Gray model was unadjusted—their magnitudes should not be compared directly. The concordant direction and statistical significance of the two analyses support the robustness of the association. Abbreviations: CI, confidence interval; CALLY, C-reactive protein–albumin–lymphocyte.

S6. Sensitivity analysis with post-stenting chemotherapy/radiotherapy as a time-fixed covariate

In the originally prespecified models, each index was adjusted for post-stenting chemotherapy/radiotherapy and stenosis site. Because chemotherapy/radiotherapy begins after baseline, modeling it as a time-fixed covariate is susceptible to immortal-time and treatment-selection bias; these models are therefore presented as a sensitivity analysis, with the primary models restricted to preprocedural covariates (Table 3 of the main text) and a time-dependent analysis presented in Table S7.

Table S6. Cause-specific Cox models with post-stenting chemotherapy/radiotherapy as a time-fixed covariate

Model	Variable	aHR	95% CI	*p*-value
**Model 1 (CALLY)**	**CALLY ≤0.110**	**15.54**	**4.85–49.81**	**<0.001**
Model 1 (CALLY)	Chemotherapy or radiotherapy	2.08	0.78–5.58	0.145
Model 1 (CALLY)	Pylorus/duodenum (*vs.* antrum)	0.91	0.33–2.51	0.863
Model 1 (CALLY)	C-index=0.823			
**Model 2 (CAR)**	**CAR >0.36**	**22.68**	**2.89–177.92**	**0.003**
Model 2 (CAR)	Chemotherapy or radiotherapy	2.65	0.99–7.07	0.051
Model 2 (CAR)	Pylorus/duodenum (*vs.* antrum)	1.12	0.41–3.06	0.825
Model 2 (CAR)	C-index=0.786			
Model 3 (mGPS)	mGPS≥1	—	—	—*
**Model 3 (mGPS)**	**Chemotherapy or radiotherapy**	**2.67**	**1.01–7.06**	**0.048**
Model 3 (mGPS)	Pylorus/duodenum (*vs.* antrum)	0.93	0.34–2.52	0.879
Model 3 (mGPS)	C-index not reported (complete separation)			
**Model 4 (NLR)**	**NLR>4.45**	**4.45**	**1.44–13.76**	**0.010**
Model 4 (NLR)	Chemotherapy or radiotherapy	1.50	0.57–3.95	0.411
Model 4 (NLR)	Pylorus/duodenum (*vs.* antrum)	0.85	0.31–2.32	0.753

Model	Variable	aHR	95% CI	*p*-value
Model 4 (NLR)	C-index=0.707			
**Model 5 (PNI)**	**PNI≤41.22**	**3.71**	**1.18–11.70**	**0.025**
Model 5 (PNI)	Chemotherapy or radiotherapy	1.75	0.66–4.66	0.262
Model 5 (PNI)	Pylorus/duodenum (*vs.* antrum)	0.88	0.32–2.41	0.803
Model 5 (PNI)	C-index = 0.684			
**Model 6 (SII)**	**SII>2759.92**	**6.39**	**2.11–19.36**	**0.001**
Model 6 (SII)	Chemotherapy or radiotherapy	1.08	0.40–2.94	0.882
Model 6 (SII)	Pylorus/duodenum (*vs.* antrum)	0.51	0.18–1.47	0.212
Model 6 (SII)	C-index=0.660			

Each model contains one dichotomized index, post-stenting chemotherapy/radiotherapy, and stenosis site (the originally prespecified specification). The CALLY estimate (aHR, 15.54; 95% CI, 4.85–49.81) was consistent with the primary preprocedural model (aHR, 12.30; 95% CI, 3.89–38.83). For mGPS ≥1, neither the adjusted Cox effect nor the model C-index is reported because of complete separation; in the unadjusted competing-risk analysis, Gray's test p = 0.002. Abbreviations: aHR, adjusted hazard ratio; CI, confidence interval.

S7. Sensitivity analysis with post-stenting chemotherapy/radiotherapy as a time-dependent covariate

To address immortal-time bias directly, post-stenting chemotherapy/radiotherapy was modeled as a time-dependent covariate that switches from 0 to 1 at the date of treatment initiation (median, 17.5 days after stenting; range, 6–30). The CALLY association was essentially unchanged, whereas chemotherapy/radiotherapy showed no association with restenosis, a pattern consistent with treatment-selection and immortal-time bias in the time-fixed models (Table S6).

Table S7. Cause-specific Cox model for tumor overgrowth restenosis with post-stenting chemotherapy/radiotherapy as a time-dependent covariate

**Variable**	**aHR**	**95% CI**	**p-value**
CALLY ≤0.110	13.59	4.20–43.98	<0.001
Chemotherapy/radiotherapy (time-dependent)	1.93	0.70–5.30	0.202
Site: Pylorus/duodenum (vs. antrum)	0.98	0.35–2.74	0.965
Stent length (per 10 mm increase)	1.15	0.91–1.46	0.234

S8. Sensitivity analyses adding tumor-related covariates

Tumor-related covariates and ECOG performance status (≥3) were added one at a time to the primary CALLY-based model (CALLY ≤0.110, stenosis site, and stent length) to limit overfitting given 17 events. The CALLY estimate remained stable across all specifications. In a further analysis, stent length was replaced with endoscopically or radiologically measured stricture length (Spearman ϱ = 0.93 with stent length). In an additional sensitivity analysis, the two patients receiving antibiotics for mild bronchopneumonia at baseline sampling were excluded; the CALLY association was essentially unchanged.

Table S8. Sensitivity analyses assessing the stability of the CALLY association

**Model**	**CALLY aHR (95% CI)**	**Added covariate aHR (95% CI)**	**p (covariate)**	**C-index**
Primary model (site + stent length)	12.30 (3.89–38.83)	—	—	0.836
+ Clinical M1	17.13 (4.77–61.48)	3.06 (0.92–10.23)	0.069	0.860
+ Peritoneal metastasis	13.96 (4.09–47.68)	1.84 (0.38–8.93)	0.451	0.837
+ PD or SRC histology	15.92 (4.56–55.66)	0.51 (0.18–1.48)	0.215	0.814
+ Prior chemotherapy	12.48 (3.93–39.64)	1.56 (0.57–4.24)	0.387	0.851
+ ECOG ≥3	11.94 (3.68–38.69)	1.15 (0.38–3.46)	0.807	0.830
Stricture length (cm) replacing stent length	13.06 (4.15–41.02)	1.19 (0.95–1.49)	0.122	0.829
Excluding two patients receiving antibiotics at sampling	16.30 (4.47–59.50)	—	—	0.857

S9. Continuous-variable analyses and tests for nonlinearity

To address the loss of information from dichotomization, each index was analyzed as a continuous variable in cause-specific Cox models adjusted for stenosis site and stent length. Right-skewed indices (CALLY, CAR, NLR, SII) were natural-log transformed; hazard ratios are expressed per one standard deviation. Nonlinearity was assessed by adding restricted cubic spline terms (4 knots at the 5th, 35th, 65th, and 95th percentiles) and comparing nested models with likelihood ratio tests. No index showed evidence of nonlinearity. The spline curve for CALLY is shown in Figure S1.

Table S9. Continuous-variable cause-specific Cox models (per one standard deviation) adjusted for stenosis site and stent length

**Index (transformation)**	**Adjusted HR per SD (95% CI)**	**p-value**	**Nonlinearity p**	**C-index**
log CALLY	0.09 (0.03–0.26)	<0.001	0.651	0.879
log CAR	11.29 (3.87–32.95)	<0.001	0.464	0.864
log NLR	2.08 (1.35–3.20)	<0.001	0.172	0.747
log SII	1.75 (1.11–2.75)	0.015	0.594	0.710
PNI	0.65 (0.37–1.12)	0.120	0.436	0.684

S10. Time-dependent AUCs accounting for death as a competing risk

Because conventional ROC analysis does not account for the timing of events or for death as a competing risk, time-dependent AUCs at 90 and 180 days were estimated using inverse-probability-of-censoring weighting, with patients who died without restenosis retained as controls. 95% confidence intervals were obtained from 1,000 bootstrap resamples. The results were consistent with the conventional ROC analysis, with CALLY and CAR showing closely comparable discrimination.

Table S10. Time-dependent AUCs for tumor overgrowth restenosis at 90 and 180 days

**Index**	**90-day AUC (95% CI)**	**180-day AUC (95% CI)**
CALLY	0.890 (0.783–0.970)	0.884 (0.786–0.966)
CAR	0.886 (0.738–0.983)	0.885 (0.773–0.970)
NLR	0.778 (0.596–0.926)	0.782 (0.632–0.903)
SII	0.707 (0.484–0.894)	0.703 (0.508–0.874)
PNI	0.638 (0.495–0.779)	0.629 (0.477–0.779)
mGPS	0.664 (0.526–0.777)	0.689 (0.563–0.799)

Figure S1. Restricted cubic spline analysis of continuous CALLY

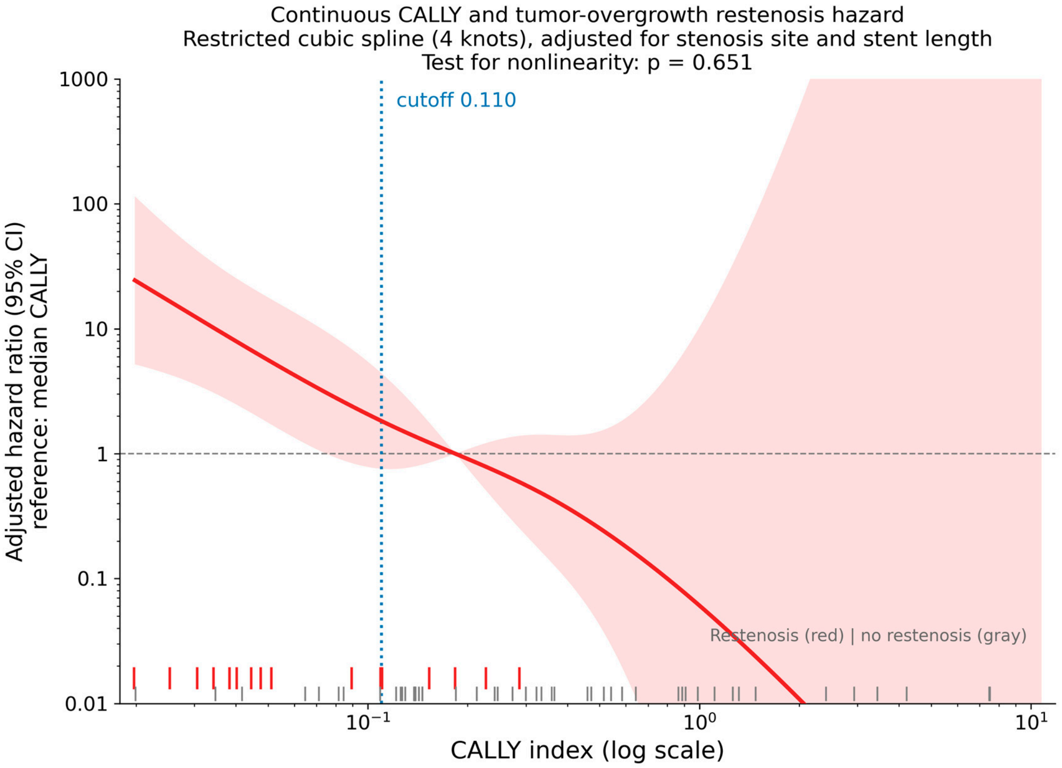

Figure S1. Adjusted hazard ratio for tumor overgrowth restenosis as a smooth function of the CALLY index (log scale), from a cause-specific Cox model with restricted cubic splines (4 knots), adjusted for stenosis site and stent length. The reference is the median CALLY value; the shaded band is the pointwise 95% CI; tick marks show individual patients (red, restenosis; gray, no restenosis). The test for nonlinearity was not significant (p = 0.651).

Figure S2. Patient selection flowchart

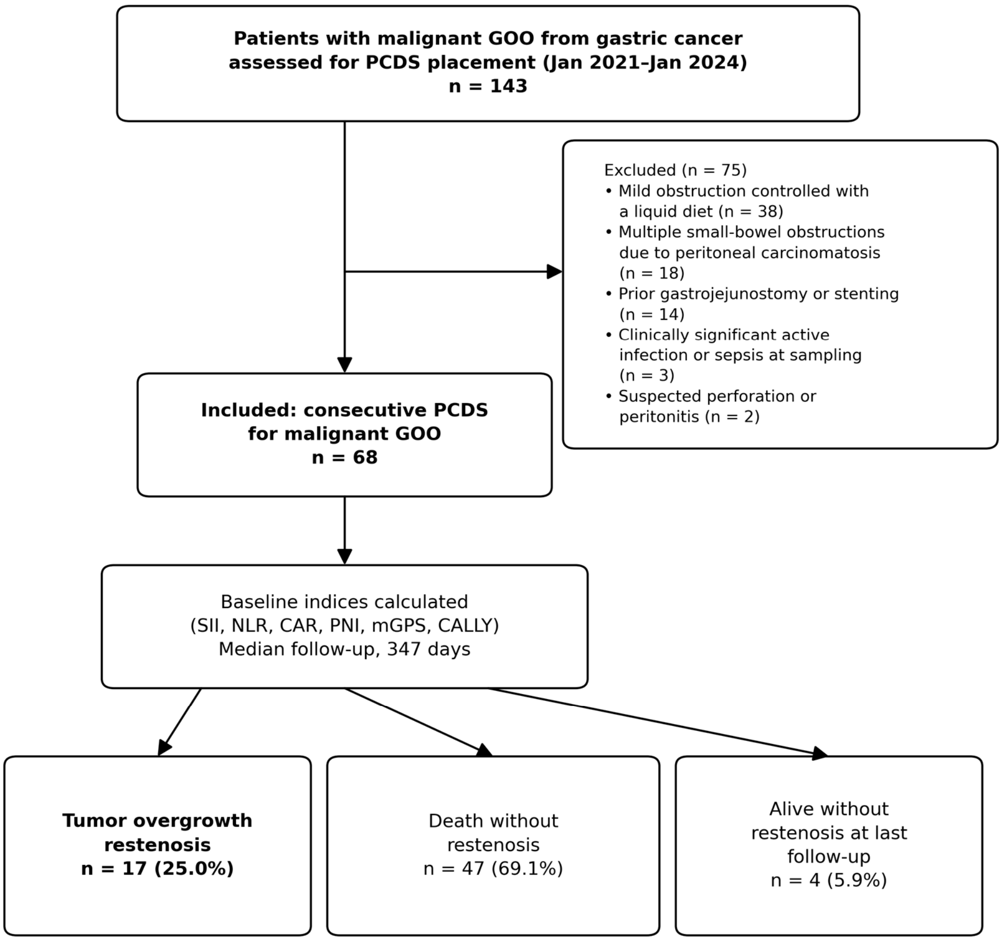

Figure S2. Patient selection flowchart. Of 143 patients with malignant gastric outlet obstruction from gastric cancer assessed for partially covered duodenal stent placement between January 2021 and January 2024, 75 were excluded (mild obstruction controlled with a liquid diet, n = 38; multiple small-bowel obstructions due to peritoneal carcinomatosis, n = 18; prior gastrojejunostomy or stenting, n = 14; clinically significant active infection or sepsis at sampling, n = 3; suspected perforation or peritonitis, n = 2), leaving 68 consecutive patients. During follow-up, 17 developed tumor overgrowth restenosis, 47 died without restenosis, and 4 were alive without restenosis at last follow-up. GOO, gastric outlet obstruction; PCDS, partially covered duodenal stent.

S11. STROBE checklist

This study is reported in accordance with the STROBE (Strengthening the Reporting of Observational Studies in Epidemiology) statement for cohort studies. The table below indicates where each checklist item is addressed in the manuscript.

Table S11. STROBE statement—checklist of items that should be included in reports of cohort studies

Section/Topic	Item No	Recommendation	Reported in
Title and abstract	1	(a) Indicate the study's design with a commonly used term in the title or the abstract<br>(b) Provide in the abstract an informative and balanced summary of what was done and what was found	Title ("A Cohort Study"); Abstract
**Introduction**			
Background/rationale	2	Explain the scientific background and rationale for the investigation being reported	Section 1, paragraphs 1–3
Objectives	3	State specific objectives, including any prespecified hypotheses	Section 1, final paragraph
**Methods**			
Study design	4	Present key elements of study design early in the paper	Section 2.1
Setting	5	Describe the setting, locations, and relevant dates, including periods of recruitment, exposure, follow-up, and data collection	Section 2.1; Section 2.2
Participants	6	(a) Give the eligibility criteria, and the sources and methods of selection of participants; describe methods of follow-up<br>(b) For matched studies, give matching criteria and number of exposed and unexposed	Section 2.1 (inclusion/exclusion); Section 2.2 (follow-up schedule)<br>Not applicable
Variables	7	Clearly define all outcomes, exposures, predictors, potential confounders, and effect modifiers. Give diagnostic criteria, if applicable	Sections 2.2 and 2.4
Data sources/measurement	8	For each variable of interest, give sources of data and details of methods of assessment (measurement). Describe comparability of assessment methods if there is more than one group	Sections 2.2–2.4
Bias	9	Describe any efforts to address potential sources of bias	Sections 2.1, 2.5; Section 4 (limitations: competing risk, immortal-time bias, optimism)

Study size	10	Explain how the study size was arrived at	Section 2.1 (all consecutive eligible patients in the study period); Section 4 (limitations)
Quantitative variables	11	Explain how quantitative variables were handled in the analyses. If applicable, describe which groupings were chosen and why	Section 2.5 (Youden index cutoffs; mGPS clinical cutoff)
Statistical methods	12	(a) Describe all statistical methods, including those used to control for confounding<br>(b) Describe any methods used to examine subgroups and interactions<br>(c) Explain how missing data were addressed<br>(d) If applicable, explain how loss to follow-up was addressed<br>(e) Describe any sensitivity analyses	Section 2.5<br>Section 2.5 (index-stratified competing-risk analyses)<br>No missing data for index components<br>Section 2.2 (censoring at death or last follow-up)<br>Section 2.5; Tables S5–S10
**Results**			
Participants	13	(a) Report numbers of individuals at each stage of study<br>(b) Give reasons for non-participation at each stage<br>(c) Consider use of a flow diagram	Section 3.1 (68 consecutive patients)<br>Section 2.1 (exclusion criteria)<br>Figure S2 (patient selection flowchart)
Descriptive data	14	(a) Give characteristics of study participants and information on exposures and potential confounders<br>(b) Indicate number of participants with missing data for each variable of interest<br>(c) Summarise follow-up time	Table 1<br>No missing data<br>Section 3.2 (median 347 days)
Outcome data	15	Report numbers of outcome events or summary measures over time	Section 3.2; Table S4
Main results	16	(a) Give unadjusted estimates and, if applicable, confounder-adjusted estimates and their precision. Make clear which confounders were adjusted for and why they were included<br>(b) Report category boundaries when continuous variables were categorized<br>(c) If relevant, consider translating estimates of relative risk into absolute risk	Tables 2, 3, S3; Section 2.5 (a priori covariates)<br>Table 2 (cutoffs)<br>Section 3.5; Table S4 (cumulative incidence)
Other analyses	17	Report other analyses done—e.g., analyses of subgroups and	Section 3.6; Tables S1, S2, S5–S10

		interactions, and sensitivity analyses	
**Discussion**			
Key results	18	Summarise key results with reference to study objectives	Section 4, paragraph 1
Limitations	19	Discuss limitations of the study, taking into account sources of potential bias or imprecision. Discuss both direction and magnitude of any potential bias	Section 4, final paragraph
Interpretation	20	Give a cautious overall interpretation of results considering objectives, limitations, multiplicity of analyses, results from similar studies, and other relevant evidence	Section 4; Section 5
Generalisability	21	Discuss the generalisability (external validity) of the study results	Section 4 (limitations); Section 5
**Other information**			
Funding	22	Give the source of funding and the role of the funders for the present study and, if applicable, for the original study on which the present article is based	Funding; Conflicts of Interest

Note: An explanation and elaboration article discusses each checklist item and gives methodological background and published examples of transparent reporting. The STROBE checklist is best used in conjunction with this article (freely available on the Web sites of PLoS Medicine at http://www.plosmedicine.org/, Annals of Internal Medicine at http://www.annals.org/, and Epidemiology at http://www.epidem.com/). Information on the STROBE Initiative is available at http://www.strobe-statement.org.
